# Targeting Mitochondria in MASLD: Comparative Evaluation of MitoQ and SS-31 (Elamipretide) in Aged Female Mice under Nutritional Stress

**DOI:** 10.33549/physiolres.935810

**Published:** 2026-08-01

**Authors:** Di XIAO, Xingxing CUI, Sajad ALI, Changfei LI, Bin WANG

**Affiliations:** 1 Department of Hepatology, Public Health Clinical Center Affiliated to Shandong University, Jinan, China; 2 Department of Biological Sciences, College of Science, King Faisal University, Al-Ahsa, Saudi Arabia

**Keywords:** MitoQ, SS-31, Mitochondrial dysfunction, Fibrosis, Non-alcoholic fatty liver disease (MASLD)

## Abstract

Metabolic Dysfunction-Associated Steatotic Liver Disease (MASLD) is a major cause of cirrhosis, liver cancer, and cardiovascular disease. Early mitochondrial dysfunction drives lipid imbalance, inflammation, and fibrosis. Given the lack of approved pharmacological treatments, this study compares the mitochondria-targeted agents MitoQ and SS-31 for their effects on mitochondrial function, oxidative stress, apoptosis, and inflammation in aged, nutritionally stressed mice with MASLD. Aged female C57BL/6 mice (12–14 months) were fed a high-fat, high-fructose diet for 16 weeks to induce MASLD. Mice were randomized into four groups: (i) control diet, (ii) MASLD, (iii) MASLD + MitoQ (25 mg/kg/day, oral), and (iv) MASLD + SS-31 (3 mg/kg/day, i.p.). Liver tissues were analyzed by western blotting for mitochondrial biogenesis and dynamics markers (PGC-1α, NRF1, and TFAM), oxidative stress regulators (SOD2, Nrf2, 4-HNE), apoptosis-related proteins (Bax, and Bcl-2), inflammatory and fibrotic mediators (NF-κB, and NLRP3), and insulin signaling proteins (p-Akt, and GLUT2). Both MitoQ and SS-31 significantly improved mitochondrial protein expression and overall liver health compared with untreated MASLD mice. MitoQ primarily enhanced mitochondrial antioxidant defenses by upregulating SOD2 and Nrf2 while decreasing 4-HNE adduct formation, reflecting reduced oxidative damage. SS-31, in contrast, more effectively preserved mitochondrial structural integrity. Both compounds attenuated inflammation by suppressing NF-κB and NLRP3 activation. They also improved insulin sensitivity by increasing p-Akt and GLUT2 expression. Histological analysis using H&E staining revealed marked reductions in hepatic steatosis. Mitochondrial dysfunction drives MASLD progression. In aged MASLD mice, MitoQ enhances antioxidant defense, SS-31 preserves integrity, and both improve insulin signaling and reduce fibrosis, supporting mitochondrial therapy for MASLD.

## Introduction

Metabolic Dysfunction-Associated Steatotic Liver Disease (MASLD) has become the most common chronic liver disorder globally, affecting almost a quarter of adults [1]. It includes a wide array of diseases, ranging from simple steatosis to non-alcoholic steatohepatitis (NASH), fibrosis, cirrhosis and hepatocellular carcinoma (HCC) [2]. MASLD is closely associated with obesity, insulin resistance, dyslipidemia, and type 2 diabetes mellitus and reflects the hepatic manifestation of the metabolic syndrome [3]. Its increasing prevalence in aging and postmenopausal women highlights the importance of studying disease mechanisms within this cohort, as aging is characterized by mitochondrial dysfunction, oxidative stress, and disrupted endocrine status that combine to exacerbate dysmetabolic response, lipid retention, and hepatic inflammation [4].

The etiopathogenesis of MASLD is multifaceted and influenced by both genetic, environmental and metabolic factors [5]. Thus, the traditional “two-hit” hypothesis—i.e., that fat deposition is a first hit and oxidative stress production is a second hit—is replaced by a model structured along multiple hits [6]. This modern perspective accepts the fact that insulin resistance, mitochondrial dysfunction, ER stress, lipotoxicity, inflammation and dysregulated adipocyte signaling are not independent processes but rather work in concert (and likely interact with each other) to drive disease progression [7] [8]. At the heart of these overlapping pathways lies mitochondrial dysfunction leading to defective energetics, fatty acid oxidation and redox homeostasis [9]. But the excessive lipid loading in MASLD saturates this system and causes incomplete fatty acid oxidation, too much reactive oxygen species (ROS) generation, and mitochondrial DNA (mtDNA) damage. Respiratory chain dysfunction and disruptions in hepatocyte bioenergetics, together with oxidative and inflammatory stress, perpetuate hepatocyte injury and fibrosis through a self-reinforcing cycle. Structural and functional mitochondrial alterations in MASLD are well documented [10].

Morphologically, hepatocyte mitochondria display swelling, cristae disruption and depolarization of the inner-membrane potential. Oxidative phosphorylation is ineffective, ATP levels are reduced, and mitochondrial fission exceeds fusion in function [7]. Mitochondrial fission and bioenergetics are dysregulated by over-fission or lack of fusion [11]. Concomitantly, reduced mitochondrial biogenesis—caused by decreased expression of peroxisome proliferator-activated receptor gamma coactivator 1-alpha (PGC-1α), nuclear respiratory factor 1 (NRF1), and mitochondrial transcription factor A (TFAM)—inhibits the formation of new, functionally competent mitochondria [12]. With defective mitophagy, these damaged organelles are not cleared efficiently, which secondarily leads to continued ROS production and pro-inflammatory signaling. Taken together, such changes result in the apoptosis of hepatocytes and bring into operation fibrogenic pathways including transforming growth factor beta 1 (TGF-β1) and α-smooth muscle actin (α-SMA), which eventually cause liver fibrosis [13]. Recent evidence places MASLD as a mitochondria-oriented disease, as mitochondrial dysfunction commonly predates the appearance of histological steatosis and inflammation [14]. Despite the increasing prevalence of MASLD, no pharmacological therapies have been approved to date [15]. The cornerstone of treatment is lifestyle modification, i.e. dietary restriction and exercise [16].

Of these interventions, MitoQ and SS-31 (elamipretide) are two well-established mitochondrial therapeutics with different yet complementary mechanisms of action. MitoQ is a coenzyme Q10 (ubiquinone) linked to a lipophilic triphenylphosphonium (TPP^+^) cation, which drives its accumulation within mitochondria in the context of their negative membrane potential [17].

In contrast, SS-31 is a cell-permeable tetrapeptide (D-Arg-dimethylTyr-Lys-Phe-NH_2_) which specifically targets cardiolipin (a mitochondria-specific phospholipid) required for preserving respiratory chain function [18]. SS-31 stabilizes the inner mitochondria membrane by interacting with cardiolipin, and decreases electron fluxes to ROS generation, thus improving ATP production efficacy. Furthermore, SS-31 has anti-apoptotic characteristics since it inhibits the release of cytochrome c and maintains mitochondrial ultrastructure [19]. Since mitochondrial oxidative damage, bioenergetic dysfunction and apoptosis are strongly related to each other in MASLD, MitoQ and SS-31 might have complementary therapeutic potential [20].

Aged female mice are also a translationally relevant model, as both age and sex greatly impact MASLD pathophysiology. Estrogen deficiency observed in post-menopausal women may worsen the lipid metabolism and development of hepatic steatosis [21]. In combination with a high-fat, high-fructose (HFHF) diet, these alterations replicate the condition of MASLD in older women where insulin resistance and metabolic inflammation mediate progression of disease [22].

In the current study, we sought to compare MitoQ with SS-31 on mitochondrial function, oxidative stress, and markers of apoptosis, inflammation and insulin resistance in aged female diet-induced MASLD. Biochemical (serum transaminases, hepatic triglycerides) and histopathological studies were also performed in order to determine the functional relationship between molecular protection and recovery of liver function. In summary, this study aims to elucidate the mechanistic foundation of mitochondria-directed therapy in MASLD, and introduces MitoQ and SS-31 as paradigms of redox-and structure-targeted mitochondrial protection.

## Methods

### Antibodies and reagents

All primary antibodies employed for western blot analyses included PGC-1α (rabbit polyclonal, Abcam, ab54481, 1:1000), NRF1 (rabbit polyclonal, Abcam, ab175932, 1:1000), and TFAM (mouse monoclonal, Cell Signaling Technology, 8076, 1:1000). Mitochondrial respiratory chain complexes were probed using antibodies against Complex I (NDUFB8, mouse monoclonal, Abcam, ab110242, 1:1000), Complex II (SDHB, mouse monoclonal, Abcam, ab14714, 1:1000), Complex III (UQCRC2, mouse monoclonal, Abcam, ab14745, 1:1000), Complex IV (MTCO1, mouse monoclonal, Abcam, ab14705, 1:1000), and Complex V (ATP5A, mouse monoclonal, Abcam, ab14748, 1:1000).

For oxidative stress and antioxidant profiling, antibodies against SOD2 (rabbit polyclonal, Cell Signaling Technology, 13141, 1:1000), Nrf2 (rabbit polyclonal, Abcam, ab62352, 1:1000), HO-1 (rabbit polyclonal, Abcam, ab13243, 1:1000), and 4-HNE (rabbit polyclonal, Abcam, ab46545, 1:500) were used. Apoptosis-related proteins were assessed using Bax (rabbit monoclonal, Cell Signaling Technology, 2772, 1:1000), Bcl-2 (rabbit monoclonal, Cell Signaling Technology, 2876, 1:1000), cytochrome c (mouse monoclonal, BD Biosciences, 556433, 1:1000), and cleaved caspase-3 (rabbit monoclonal, Cell Signaling Technology, 9661, 1:1000).

Inflammatory markers were detected using NF-κB (rabbit polyclonal, Cell Signaling Technology, 8242, 1:1000) and NLRP3 (rabbit monoclonal, AdipoGen, AG-20B-0014, 1:1000), whereas fibrotic proteins were examined using α-SMA (mouse monoclonal, Sigma-Aldrich, A5228, 1:1000) and TGF-β1 (rabbit polyclonal, Abcam, ab92486, 1:1000). To evaluate insulin signaling, antibodies against p-IRS1 (Tyr612, rabbit monoclonal, Cell Signaling Technology, 2381, 1:1000), p-Akt (Ser473, rabbit monoclonal, Cell Signaling Technology, 4060, 1:1000), and GLUT2 (rabbit polyclonal, Abcam, ab54460, 1:1000) were used. GAPDH (mouse monoclonal, Sigma-Aldrich, A5316, 1:5000) served as the internal loading control. Secondary antibodies for Western blot included HRP-conjugated goat anti-rabbit IgG (Abcam, ab6721, 1:5000) and HRP-conjugated goat anti-mouse IgG (Abcam, ab6789, 1:5000).

Mitochondria-targeted therapeutics included MitoQ (Cayman Chemical), administered orally at 25 mg/kg/day, and SS-31 (Elamipretide) (MedChem-Express), administered intraperitoneally at 3 mg/kg/day. Additional reagents used were RIPA lysis buffer (Thermo Fisher, 89900), protease and phosphatase inhibitor cocktail (Sigma-Aldrich, P8340), BCA protein assay kit (Thermo Fisher, 23225), TBARS assay kit (Cayman Chemical, 10009055), TUNEL assay kit (Roche, 11684795910), and staining reagents including hematoxylin and eosin (Sigma-Aldrich, HHS32), Masson’s trichrome (Sigma-Aldrich, HT15), Sirius Red (Abcam, ab150681), and DAB substrate kit (Vector Labs, SK-4100).

### Animals and experimental design

Aged female C57BL/6 mice (12–14 months old, 25–30 g) were obtained from the Institutional Animal Facility and acclimatized for one week under controlled conditions (22 ± 2 °C, 55 ± 5% humidity, 12 h light/dark cycle) with free access to food and water. All procedures were approved by the Ethics Committee of Public Health Clinical Center Affiliated to Shandong University (approval number: GWLCZXEC-SOP-K-2026-20) and were conducted in accordance with institutional guidelines for the care and use of laboratory animals, with careful attention to minimizing stress and ensuring humane treatment.

To model diet-induced MASLD, mice were fed a high-fat, high-fructose (HFHF) diet for 16 weeks (60% kcal from fat and 30% fructose in drinking water) [23]. Control animals were fed with the standard chow diet (10% kcal from fat). After 8 weeks of diet feeding, animals were randomly divided into four groups (n = 8 per group). After 8 weeks of diet feeding, animals were randomly divided into four groups (n = 8 per group):

1. Control (standard chow)
2. MASLD (HFHF diet)
3. MASLD + MitoQ (25 mg/kg/day, oral)
4. MASLD + SS-31 (3 mg/kg/day, ip)

The doses were selected based on previous studies demonstrating improvement in mitochondrial function. Treatment was maintained over 8 weeks along with the HFHF diet. Body weight and food intake were recorded weekly to assess physiological and metabolic changes.

### Sample collection and tissue processing

At the end of 16 weeks, mice were fasted overnight (12 hours) and anesthetized with ketamine (80 mg/kg) and xylazine (10 mg/kg). Blood was taken by cardiac puncture for serum preparation and kept at −80 °C; livers were removed, weighed, and partitioned for histology, molecular assays, and microscopy. Liver-to-body weight ratios were calculated. Samples for histology were fixed in 10% neutral-buffered formalin (24–48 h) and snap-frozen for molecular analyses in liquid-nitrogen before storage at −80 °C.

### Biochemical assays

Liver injury was determined by measuring serum levels of ALT and AST with commercial kits (Erba Diagnostics, India) [24]. Hepatic triglycerides were dissolved (chloroform–methanol, 2:1 v/v) and determined by colorimetry with reagents of Sigma-Aldrich, USA. The protein concentration was estimated with the BCA assay.

Levels of the oxidative stress markers MDA was assessed according to TBARS assay [25] and antioxidant defenses (SOD, catalase, GSH) measured by spectrophotometry and normalized against total proteins [26].

### Histopathological evaluation

Paraffin-embedded tissues were sectioned at 5 μm and stained with H&E for hepatocytes and Masson's trichrome for fibrosis. Slides were blindly scored with the MASLD Activity Score (NAS; 0–8) and fibrosis stage (0–4) by two independent pathologists [27].

### Western Blot analysis

Frozen liver pieces were homogenized in lysis buffer (RIPA) containing inhibitors; after centrifugation (12,000 × g, 15 min, 4 °C), the supernatant was removed. Proteins (30–40 μg) were separated by SDS−polyacrylamide gel electrophoresis, transferred to Polyvinylidene fluoride membranes, blocked (5% milk/TBST), and then incubated overnight at 4 °C with primary antibodies directed against mitochondrial, oxidative, apoptotic, inflammatory, fibrotic and insulin signaling/transduction signaling proteins. Blots were probed with HRP-conjugated secondary antibodies, visualized by ECL, and quantified using ImageJ, normalized to β-actin.

### Assessment of insulin signaling

Hepatic insulin signaling was evaluated *via* Western blot for p-IRS1, p-Akt, and GLUT2. Fasting glucose was measured using a glucometer, and insulin levels determined by ELISA (Crystal Chem, USA). Insulin resistance (HOMA-IR) was calculated as:


HOMA-IR=(Fasting glucose (mmol/L)×Fasting   insulin (μU/mL)/22.5

### Statistical analysis

All experiments were conducted in triplicate. For animal studies, n = 8 per group; triplicates refer to technical replicates for in vitro assays such as western blot. Values are presented as mean ± SEM. Statistical significance between groups was determined with one-way ANOVA followed by Tukey’s post hoc test. Normality of data and homogeneity of variance were tested before analysis. Differences were considered to be significant at p < 0.05. All the analyses as well as graphs were performed with GraphPad Prism 11.0 (GraphPad Software, USA).

## Results

### MitoQ and SS-31 improve liver function and attenuate hepatic steatosis

**Fig. 1:**
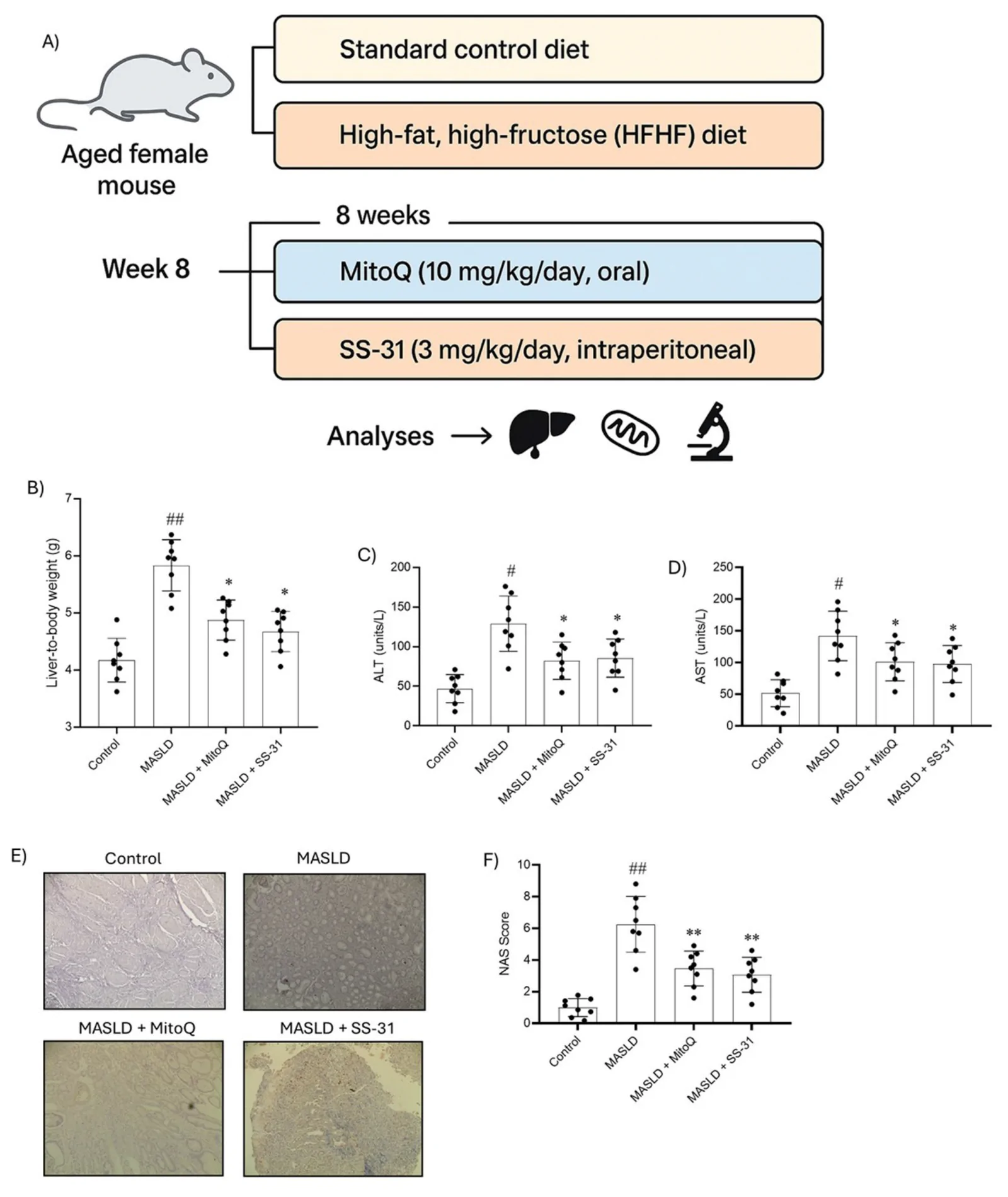
MitoQ and SS-31 attenuate hepatic dysfunction and steatosis. (**A**) Experimental design and treatment overview. (**B**) Quantification of liver to body weight ratios illustrate a clear decrease in hepatic mass following treatment. (**C–D**) Serum levels of AST and ALT in the mice are shown to improve hepatocellular injury. (**E**) H&E-stained liver sections observed extensive macrovesicular steatosis, hepatocyte ballooning, and inflammatory foci in MASLD mice, which were obviously ameliorated by treatment with both compounds. (**F**) Quantitative analysis of MASLD Activity Score (NAS) shows reduction of steatosis and inflammation. Data are presented as mean ± SEM (n = 8 animals per group). Experiments were performed in technical triplicates where applicable. #p < 0.05, and ##p < 0.01 vs control, whereas p < 0.05, *p < 0.01 vs MASLD.

**Fig. 2:**
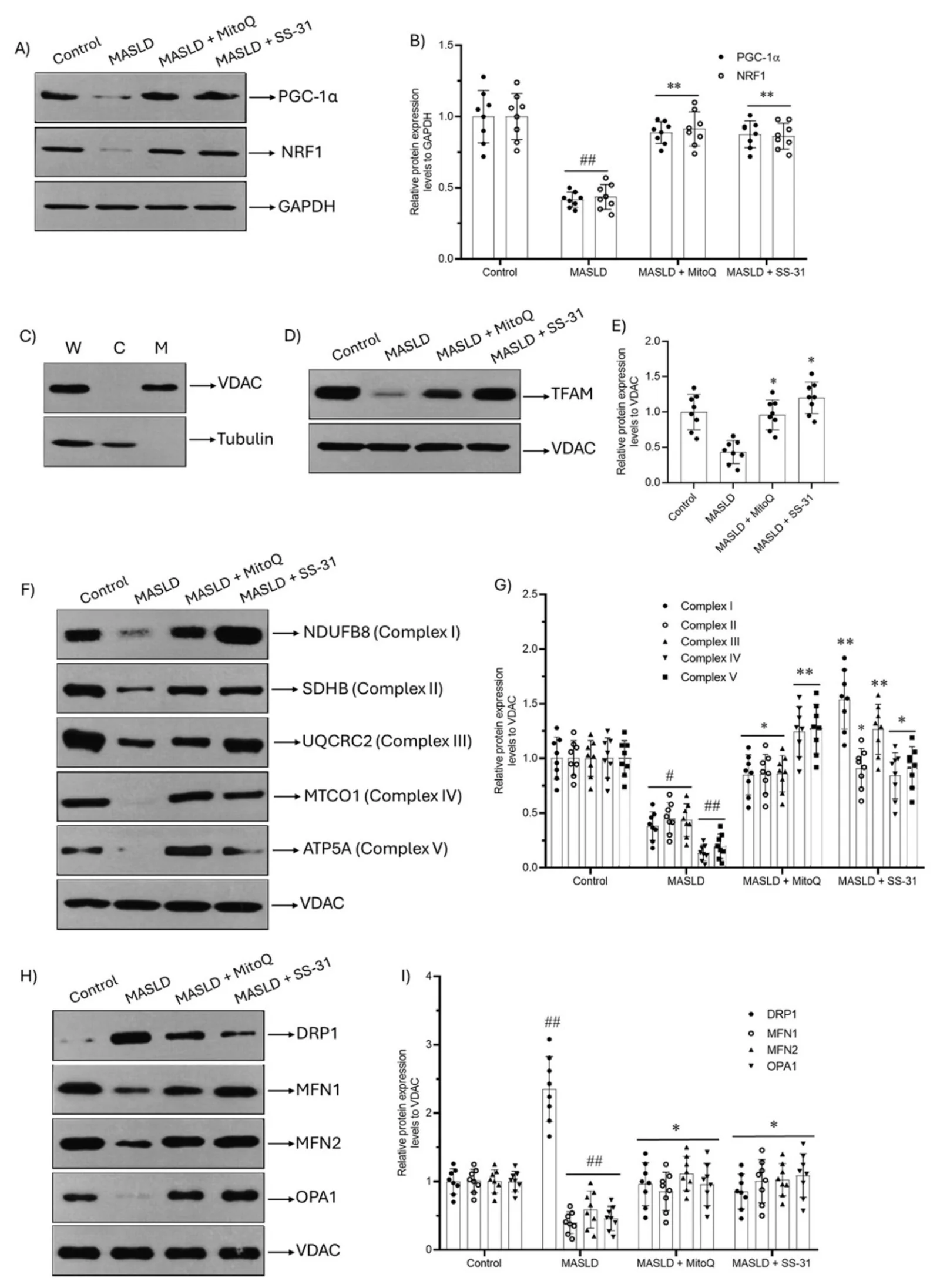
MitoQ and SS-31 restore mitochondrial biogenesis, respiration, and dynamic balance in MASLD livers. (**A**) Western blots showing expression of mitochondrial biogenesis regulators PGC-1α, and NRF1, in whole-cell lysates. Both treatments restored the reduced protein levels seen in MASLD livers. (**B**) Quantitative densitometric analysis of PGC-1α, and NRF1. (**C**) Purity of mitochondrial fractions confirmed by western blots using VDAC (mitochondrial marker) and tubulin (cytosolic marker). (**D**) Western blots showing expression of mitochondrial biogenesis regulators TFAM, in mitochondrial fraction. Both treatments restored the reduced protein levels seen in MASLD livers. (**E**) Quantitative densitometric analysis of TFAM. (**F**) Expression profiles of electron transport chain (ETC) complexes I–V, demonstrating recovery of oxidative phosphorylation proteins, with SS-31 showing stronger restoration of Complex IV and V. (**G**) Quantitative densitometric analysis of complexes I–V. (**H**) Western blots of mitochondrial dynamics proteins (DRP1, MFN1, MFN2, and OPA1) showing that MitoQ and SS-31 corrected the fission–fusion imbalance observed in MASLD. (**I**) Quantitative densitometric analysis of mitochondrial dynamics proteins. Data are presented as mean ± SEM (n = 8 animals per group). Experiments were performed in technical triplicates where applicable. #p < 0.05, and ##p < 0.01 vs. control, while as *p < 0.05, **p < 0.01 vs. MASLD.

Chronic feeding of 12–14-month-old female mice with an HFHF diet for 16 weeks was associated with pronounced hepatomegaly and disrupted metabolism. The experimental protocol and the overall treatment schedule of this model are shown in Fig. 1A. The liver/body weight ratio of MASLD mice was markedly higher than that of age-matched control (5.8 ± 0.3 % vs. 4.1 ± 0.2 %, p < 0.01), which suggested the formation of massive hepatic lipid droplets and induced steatosis in livers (Fig. 1B). Both MitoQ and SS-31 treatments significantly reduced the liver-to-body weight ratio (MitoQ: 4.9 ± 0.2 %, SS-31: 4.7 ± 0.2 %; *p* < 0.05 vs. MASLD), indicating hepatoprotection (Fig. 2A). Correspondingly, serum alanine aminotransferase (ALT) (Fig. 1C) and aspartate aminotransferase (AST) (Fig. 1D) levels increased markedly (ALT: 125 ± 10 U/L; AST: 142 ± 12 U/L vs. control ALT: 45 ± 5 U/L, AST: 52 ± 6 U/L; *p* < 0.01), confirming hepatocellular injury. As compared to the MASLD group, levels of serum ALT and AST were significantly decreased in response to both MitoQ and SS-31 treatment, reflecting liver protective effects. Both treatments did not modulate any of these parameters in control fed animals, confirming minimal off-target toxicity.

Histological evaluation by hematoxylin and eosin (H&E) staining revealed extensive macrovesicular steatosis, hepatocyte ballooning, and inflammatory infiltration in MASLD livers, predominantly around periportal and pericentral zones (Fig. 1E). The MASLD Activity Score (NAS) increased to 6.2 ± 0.4 in HFHF-fed mice from 1.0 ± 0.2 in control mice (p < 0.01). MitoQ and SS-31 caused NAS to drop significantly to 3.6 ± 0.3 and 3.3 ± 0.2, respectively (p < 0.01 vs MASLD), indicating improvement of steatosis and inflammation (Fig. 2F). SS-31 was slightly more beneficial than MitoQ, in particular for treating hepatocyte ballooning and inflammatory infiltration.

### Mitochondrial biogenesis and dynamics are enhanced

Western blotting analysis of whole cell lysates and mitochondrial enriched fractions showed that mitochondrial biogenesis was significantly suppressed in livers of MASLD mice. Expression of major regulators proteins PGC-1α and NRF1 was decreased in total cell lysates (p < 0.01), indicating an impairment in transcriptional activation of mitochondrial transcripts. The decreases in PGC-1α and NRF1 caused by HFHF diet were normalized after treatment with MitoQ or SS-31 to levels close to the control group (p < 0.01) (Fig. 2 A–B).

In line with this recovery pattern, the mitochondrial transcription factor TFAM (required for replication and transcription of mtDNA) was also markedly reduced in MASLD livers but fully restored after treatment. TFAM was also enhanced about 2.1- and 2.4-fold in MitoQ and SS-31-treated MASLD mice respectively versus untreated one (p < 0.05) (Fig. 2D–E), demonstrating both compounds induce organelle-level mitochondrial biogenesis as well.

The protein levels of the electron transport chain (ETC) complexes I–V were measured in mitochondrial fractions to evaluate mitochondrial function. In MASLD livers, a significant decrease in expression of ETC components was observed, suggesting impaired activity of oxidate phosphorylation and ATP synthesis. Levels of both MitoQ and SS-31 restored ETC protein content in a significant manner, supporting better mitochondrial respiratory capacity. Notably, treatment resulted in a more pronounced restoration of Complex IV and Complex V subunit proteins, both of which are essential for ATP synthesis and global mitochondrial energetics (Fig. 2F–G).

We then sought to determine whether these functional responses were associated with altered mitochondrial dynamics. A profound alteration of the mitochondrial fusion-and-fission balance characterized by upregulation of a fission marker (DRP1) and downregulation of fusion markers (MFN1, MFN2, OPA1)—indicating excessive dismemberment—was evident in MASLD livers. The imbalance was rectified by MitoQ and SS-31 treatment, down-regulation of DRP1 levels, recovery of MFN1/2 and OPA1 expression for promoting mitochondrial fusion as well as preserving the integrity of mitochondria. Of the two, SS-31 yielded a relatively better enhancement of protein bands associated with fusion in relation to its more potent effect on mitochondrial respiration and ATP homeostasis (Fig. 2H–I).

Together these data demonstrate that MitoQ and SS-31 oppose the development of MASLD-induced mitochondrial dysfunction by stimulating biogenesis, increasing respiratory quality, and restoring a positive balance between fission and fusion.

### Mitochondrial oxidative stress is attenuated

**Fig. 3:**
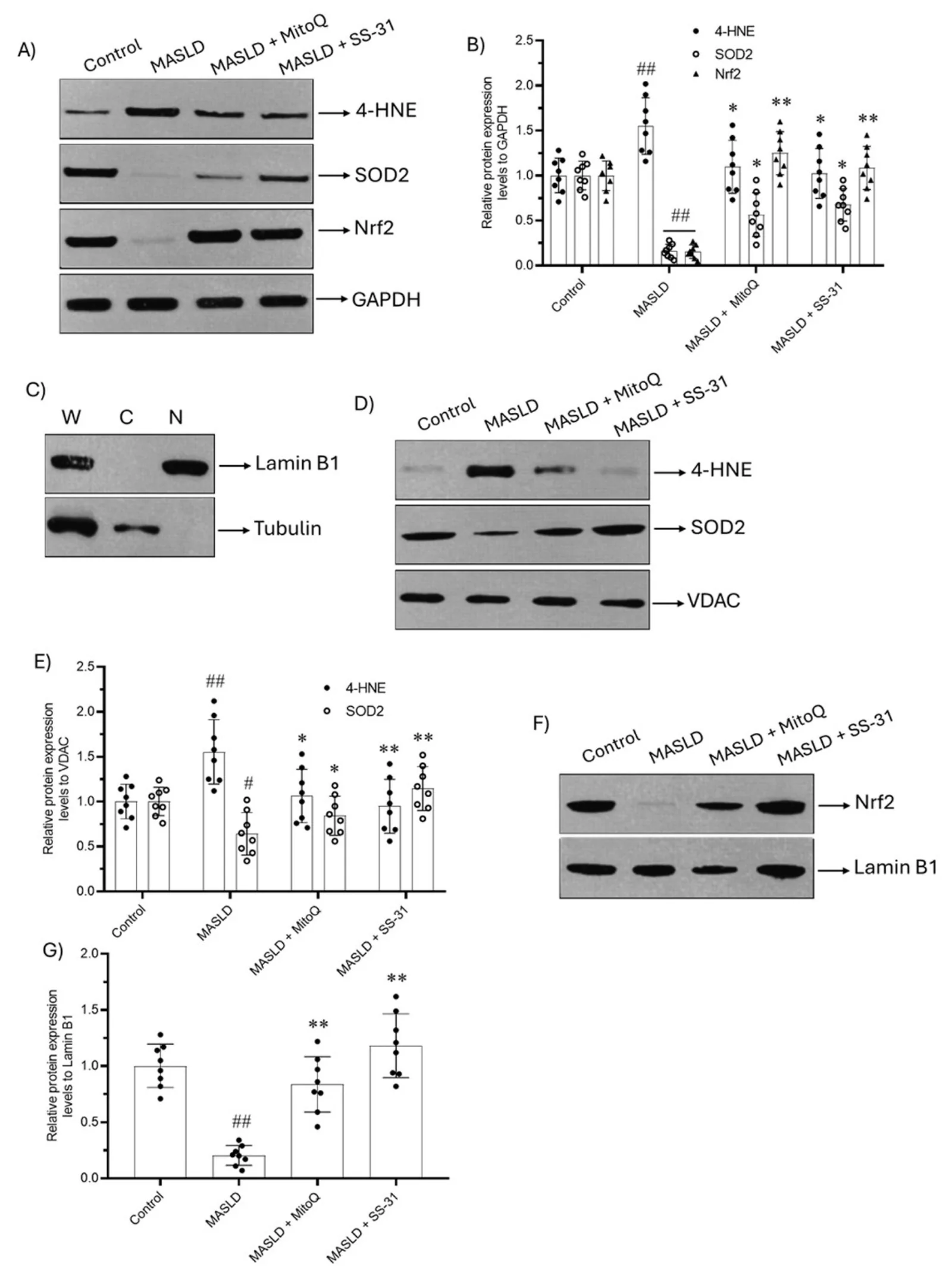
MitoQ and SS-31 alleviate Mitochondrial Oxidative Stress via complementary mechanisms. (**A**) Western blots of the 4-HNE, SOD2 and Nrf2 in whole-cell lysate. (**B**) Densitometric quantitative analysis of 4- HNE, SOD2 and Nrf2 (**C**) Validation of subcellular fraction purity using Lamin B1 (nuclear marker). (**D**) Western blots showing expression levels of 4-HNE, SOD2, and Nrf2 levels in nuclear cell lysate. (**E**) Quantitative densitometric analysis of 4-HNE, SOD2, and Nrf2. (**F**) Western blot shows that MASLD reduced Nrf2 accumulation in nucleus, while MitoQ and SS-31 reversed these changes. (**G**) Quantitative densitometric analysis of Nrf2. Data are presented as mean ± SEM (n = 8 animals per group). Experiments were performed in technical triplicates where applicable. #p < 0.05, and ##p < 0.01 vs. control, while as *p < 0.05, **p < 0.01 vs. MASLD.

Livers from MASLD mice showed clear signs of oxidative stress, reflected by elevated 4-hydroxy-2-nonenal (4-HNE) adducts and reduced antioxidant proteins. Western blotting on whole-cell lysates showed marked 4-HNE accumulation and reduced SOD2 and Nrf2 levels (p < 0.01). A strong recovery of SOD2 and Nrf2 and a significant reduction in 4-HNE content were observed after MitoQ treatment (Fig. 3A–B).

To determine the location where these alterations occur, nuclear fractions were prepared, and tested for purity using Lamin B1 (nuclear marker) and tubulin (cytoplasmic marker), and verified lack of cross-contamination (Fig. 3C).

MASLD livers also showed a significant oxidative damage in nuclear cell, observed by 4-HNE upregulation and lowered levels of SOD2. MitoQ treatment largely rescued mitochondrial SOD2 protein levels (by 2.5-fold, p < 0.01), indicative of robust mitochondrial anti-oxidant protection against insult. SS-31 also decreased 4-HNE levels (p < 0.01) and modestly increased in SOD2 expression, albeit to a lesser extent than MitoQ (Fig. 3D–E).

Subcellular distribution studies in nuclear fractions revealed that MASLD considerably reduced the nuclear content to Nrf2, corresponding with diminished activation of antioxidant responses. Nonetheless, both treatments with MitoQ and SS-31 resulted in increased nuclear amount of Nrf2 (2.2-fold and 1.6-fold, respectively; p < 0.01), indicating reactivation of Nrf2-driven antioxidant signaling (Fig. 3F–G).

Taken together, these data suggest that MitoQ and SS-31 act by complementary mechanisms to mitigate mitochondrial oxidative stress—MitoQ mainly resetting mitochondrial redox homeostasis and SS-31 enhancing the Nrf2-mediated transcriptional defense system to reinforce macro antioxidant capacity.

### Apoptosis is suppressed by MitoQ and SS-31

**Fig. 4:**
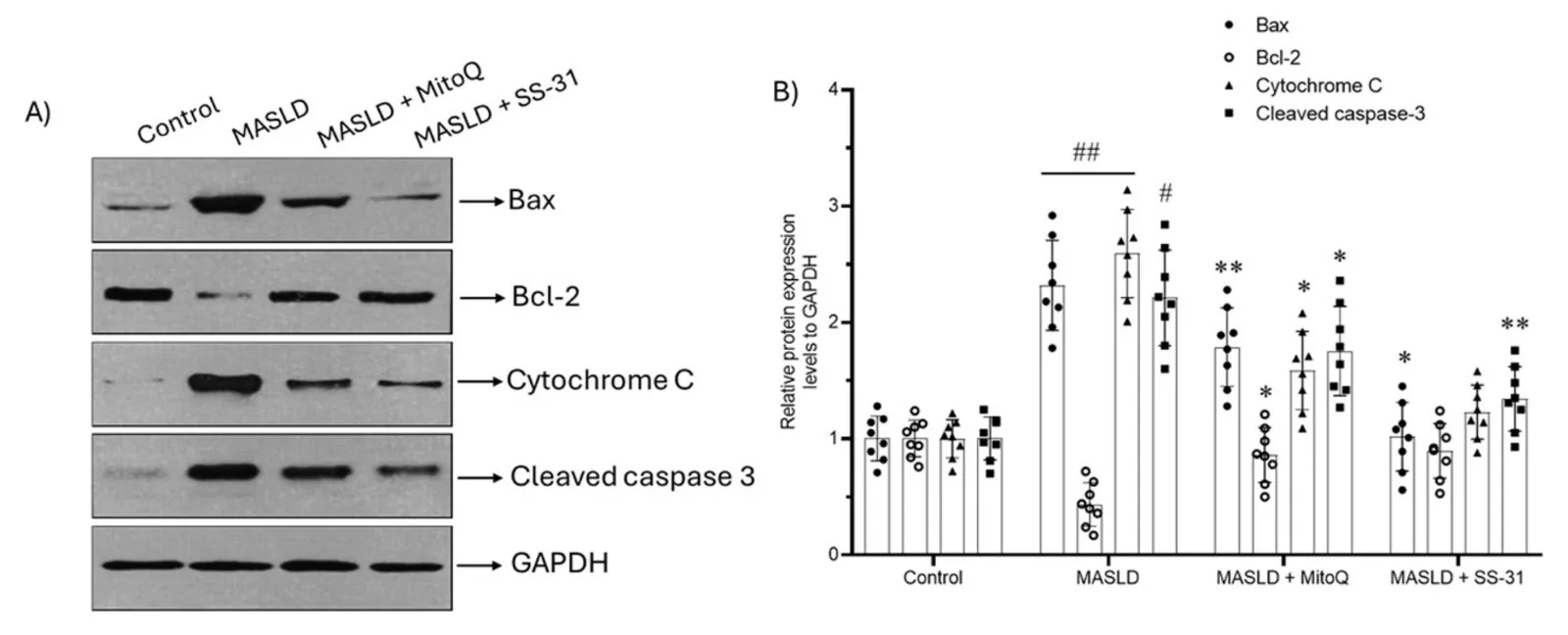
MitoQ and SS-31 attenuate hepatocyte apoptosis during MASLD. (**A**) Western blot bands of hepatic Bax, Bcl-2, cytochrome c and cleaved caspase-3 expression in control, MASLD and treatment groups. (**B**) Quantitative densitometric analysis of Bax, Bcl-2, cytochrome c and cleaved caspase-3. Data are presented as mean ± SEM (n = 8 animals per group). Experiments were performed in technical triplicates where applicable. #p < 0.05, and ##p < 0.01 vs. control, p < 0.05, *p < 0.01 vs. MASLD.

Livers from MASLD mice exhibited a strong pro-apoptotic signature, indicating extensive hepatocellular injury. Western blot analyses with whole-cell lysates indicated 3.2-fold higher expression of Bax, a 2-fold decrease in Bcl-2, 2.8-fold higher cytochrome c expression and significant accumulation of cleaved caspase-3 as compared to control livers (Fig. 4A). Quantitative analysis indicated that the expression of Bax, cytochrome c and cleaved caspase-3 were significantly increased, while Bcl-2 was decreased in MASLD group as compared with control group, MitoQ plus SS-31 markedly reversed the above changes. SS-31 showed a slightly enhanced anti-apoptotic effect like that of its recognized activity in maintaining the integrity of the mitochondrial membranes (Fig. 4B). These alterations correspond to an increased susceptibility of mitochondrial membrane permeabilization and activation of the intrinsic apoptotic cascade.

MitoQ or SS-31 treatment significantly attenuated these effects. The expressions of Bax were reduced and the expression of Bcl-2 was increased in a statistically significant manner in both cases as well as cytochrome c and caspase-3 cleavage, confirming that apoptosis was strongly inhibited. MitoQ predominantly attenuated upstream pro-apoptotic signaling by stabilizing redox homeostasis, whereas SS-31 more effectively preserved mitochondrial integrity, thereby preventing cytochrome c efflux and caspase activation.

Taken together, these findings indicate that MitoQ and SS-31 exert similar effects to prevent hepatocyte apoptosis in MASLD via the regulation of mitochondrial homeostasis and oxidative stress.

### Inflammation and fibrosis are mitigated

**Fig. 5:**
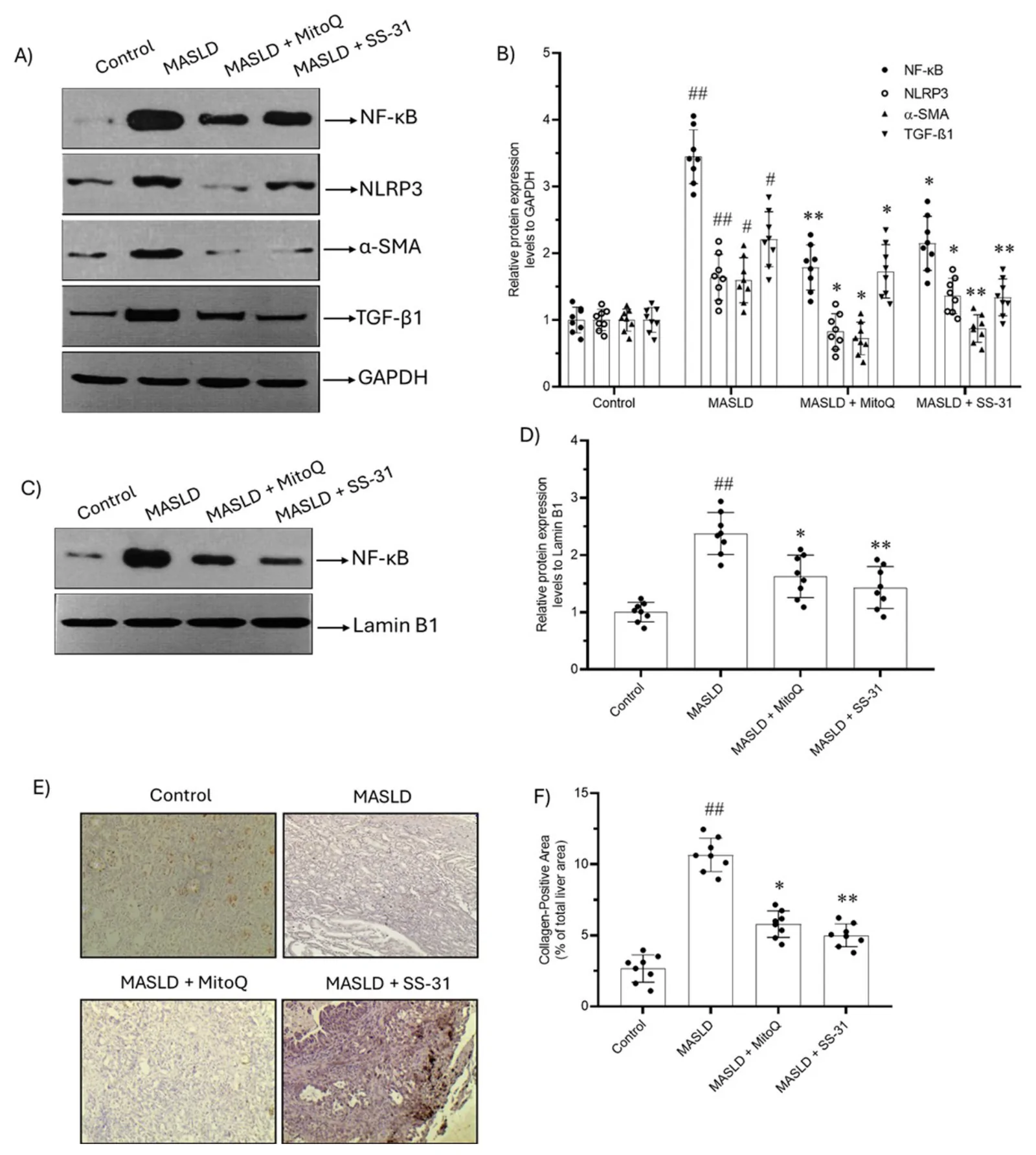
MitoQ and SS-31 alleviate hepatic inflammation, and fibrosis in MASLD. (**A**) Representative Western blots for the expression of inflammatory (NF-κB, NLRP3), and fibrotic (α-SMA, TGF-β1) markers in whole cell lysates of control, MASLD, and treatment groups. (**B**) Densitometric analysis of NF-κB, NLRP3, α-SMA and TGF-β1 was performed. (**C**) NF-κB in nuclear fractions were evaluated to determine nuclear translocation and Lamin B1. (**D**) Densitometric analysis of NF-κB as quantified to Fig. 6C. (**E**) Masson’s trichrome stained liver sections show large amounts of collagen accumulation in MASLD mice, but this was significantly inhibited by MitoQ and SS-31 treatment. (**F**) Quantitative morphometry of collagen-positive areas showing a significant reduction by both compounds, although SS-31 shows greater decrease. Data are presented as mean ± SEM (n = 8 animals per group). Experiments were performed in technical triplicates where applicable. #p < 0.05, and ##p < 0.01 vs. Control, while as p < 0.05, *p < 0.01 vs. MASLD.

In accordance with the metabolic stress caused by long term HFHF feeding, MASLD livers displayed strong induction of inflammation and fibrotic remodeling. The western blotting results of levels of proinflammatory and fibrogenic responses factors, including NF-κB, NLRP3, α-SMA and TGF-β1 in whole cell lysate were observably upregulated (Fig. 5A). NF-κB and NLRP3 levels were up by 3.5 and 3.1-fold (p < 0.01), respectively, whereas α-SMA and TGF-β1 up-regulated by 3.8 and 3.5-fold (p < 05) (Fig. 5B), illustrating the development from fatty liver to steatohepatitis with active fibrosis. MitoQ and SS-31 both significantly abrogate overactivation of inflammatory and fibrotic pathways. MitoQ predominantly attenuated NF-κB and NLRP3 expression, whereas SS-31 had slightly stronger inhibitory effect on α-SMA and TGF-β1, indicative of a synergistic protective action.

NF-κB induction at the transcriptional level was verified by separating nuclear and cytosolic fractions. HFHF diet-fed mice exhibited marked nuclear accumulation of NF-κB p65, indicative of its activation and translocation to the nucleus. Treatment with MitoQ and SS-31 effectively abolished the increased nuclear translocation of NF-κB p65, thereby preserving its normal localization and inhibiting NF-κB–driven inflammatory gene expression (Fig. 5C–D).

These molecular observations were corroborated by histological data: analysis of liver sections from mice fed a HFHF diet showed extensive deposition of collagen surrounding hepatocytes and throughout pericentral areas, suggestive of severe fibrosis as revealed by Masson trichrome staining. MitoQ and SS-31 treatment significantly attenuated collagen deposition and fibrosis severity (Fig. 5E). Quantitative morphometric analysis revealed a 45% decrease in the collagen-positive area in MitoQ-treated mice and a 52% decrease in the SS-31 group compared with untreated MASLD mice (Figure 5F). Taken together, these results suggest that mitochondrial-targeted treatments effectively inhibit inflammatory and fibrotic advancements in MASLD and identified SS-31 with a limited improved antifibrotic activity.

### Insulin signaling and glucose homeostasis are improved

**Fig. 6:**
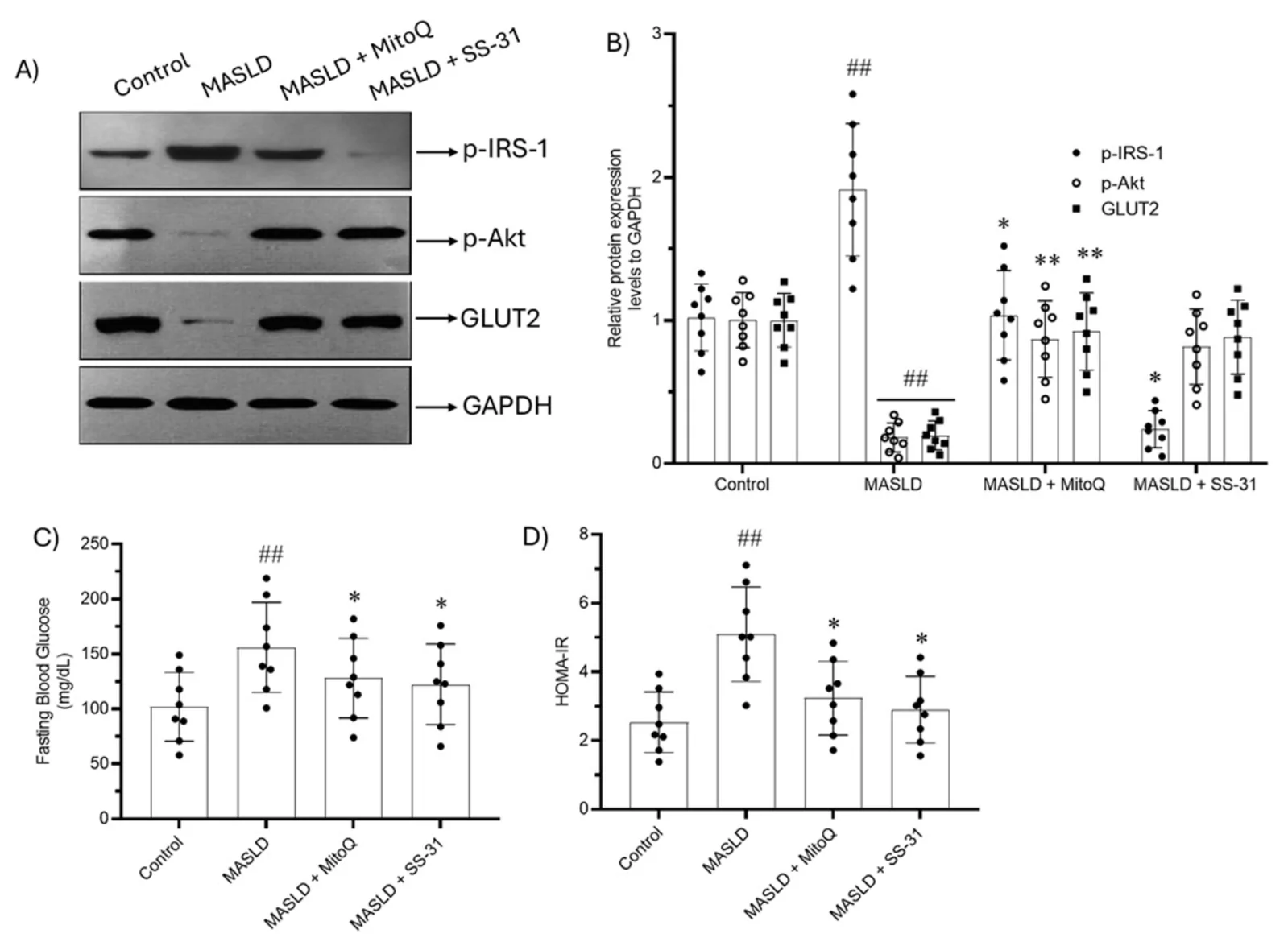
MitoQ and SS-31 improve insulin signaling and glucose metabolism. (**A**) Western blot analysis and densitometry of p-IRS1, p-Akt, and GLUT2 show recovery of insulin signaling in the treated animals. (**B**) Quantitative morphometric analysis of p-IRS1, p-Akt and GLUT2. (**C**) Fasting blood glucose and (**D**) HOMA-IR indicative of enhanced insulin sensitivity and glucose control. SS-31 exerted a modestly stronger impact on p-Akt activation and glucose correction relative to MitoQ. Data are presented as mean ± SEM (n = 8 animals per group). Experiments were performed in technical triplicates where applicable. ##p < 0.01 vs. control; as p < 0.05, *p < 0.01 vs. MASLD.

Hepatic insulin resistance, which is a signature of metabolic dysfunction in MASLD, was pronounced under chronic HFHF feeding. Western blot analysis of whole-cell liver lysates showed a marked decrease in insulin receptor substrate-1 phosphorylation (p-IRS-1) and Akt phosphorylation (p-Akt), besides lowered glucose transporter GLUT2 expression (p < 0.01; Fig. 6A–B). These molecular defects suggest that insulin signaling is suppressed and glucose uptake inhibited in hepatocytes.

These changes were largely restored by the mitochondria-targeted antioxidants MitoQ and SS-31. Both agents increased the expression of p-IRS-1, and p-Akt and the glucose transporter GLUT2 compared with those observed in MASLD mice (p < 0.05), restoring these levels toward those seen in control mice, thereby improving insulin receptor signaling and enhancing glucose transport capacity. Interestingly, SS-31 induced a marginally more profound induction of Akt phosphorylation, implying greater effects on the downstream signaling replenishing glucose utilization and metabolic recovery.

These molecular effects were manifested in distinct physiological changes. Fasting blood glucose levels, which rose sharply to 156 ± 12 mg/dL in HFHF-fed MASLD mice (versus 102 ± 8 mg/dL in controls; were significantly reduced following treatment—128 ± 10 mg/dL with MitoQ and 122 ± 9 mg/dL with SS-31 (Fig. 6C). Likewise, homeostatic model assessment for insulin resistance (HOMA-IR) increased to 5.1 ± 0.3 in MASLD animals and fell to 3.2 ± 0.3 with MitoQ and 2.9 ± 0.2 by SS-31 treatment protocol (Fig. 6D), suggesting an impressive restoration of systemic insulin sensitivity.

Collectively, these results indicate that MitoQ and SS-31 ameliorate insulin signaling in the liver and glucose homeostasis impaired by HFHF feeding. The salutary actions of these agents are probably acquired *via* suppression of oxidative stress and restoration of mitochondrial integrity, leading to homoeostasis to insulin action at cellular as well as systemic levels.

## Discussion

Metabolic Dysfunction-Associated Steatotic Liver Disease (MASLD) is a growing epidemic worldwide, particularly in the elderly population due to metabolic distress and mitochondrial dysfunction that expedite disease progression. This work highlights that the mitochondria-targeted drugs MitoQ and SS-31 (Elamipretide) rescue diet-induced MASLD in old female mice. *Via* shared mechanisms, they mediate repair of mitochondria, reduced oxidative stress, suppressed apoptosis and inflammation, restricted fibrosis, as well as enhanced insulin signaling. These findings are consistent with the growing body of literature identifying mitochondrial dysfunction as a central pathogenic driver of MASLD progression rather than merely a secondary consequence of lipid accumulation [28]. Previous experimental studies using mitochondrial antioxidants and redox-modulating agents have similarly demonstrated improvements in steatosis, oxidative injury, and metabolic homeostasis, supporting the concept that mitochondria-directed therapies may provide mechanistically relevant disease modification [29].

Our observations emphasize a key role of mitochondria in the pathogenesis and development of MASLD. Mice that consumed a high-fat, high-fructose (HFHF) diet presented with clear mitochondrial damage—they were swollen, had disrupted cristae and the mitochondria were fragmented—which shows that their energy production capabilities collapsed and they became more prone to oxidative stress. Primary regulators of mitochondrial biogenesis (PGC-1α, NRF1 and TFAM), as well as members of the electron transport chain were depleted indicating dysfunction in oxidative phosphorylation and energy production. The results are in line with previous findings that mitochondrial impairments arise in early states of MASLD, the liver damage and insulin resistance being driven by mitochondrial disease rather than fat deposition and inflammation [30]. Comparable mitochondrial ultrastructural abnormalities, including swelling, cristae loss, impaired β-oxidation, and reduced respiratory capacity, have been repeatedly described in both dietary and genetic models of steatotic liver disease [31]. Alterations in PGC-1α/NRF1/TFAM signaling have also been reported in experimental MASLD, reinforcing the view that impaired mitochondrial biogenesis is a conserved feature of disease progression [32].

MitoQ or SS-31 treatment successfully rescued mitochondrial architecture and function, whereas they worked through the different but coexistent ways. MitoQ, a mitochondria-targeted antioxidant, enhanced the endogenous anti-oxidative defense by increasing the expression of SOD2 and Nrf2 while reducing 4-HNE adducts—a major product of lipid peroxidation and oxidative damage. By attenuating the accumulation of ROS, MitoQ presumably interrupts oxidative signaling cascades driving inflammation, cell death and fibrogenesis. In SS-31-treated livers, however, the structure of mitochondrial was maintained mainly by preserving cardiolipin-enriched inner membranes leading to inhibition cytochrome c release and caspase activation. This protection at a structural level correlated with an even greater anti-apoptotic effect and demonstrated that SS-31 is capable of preserving mitochondrial integrity in the setting of metabolic stress. These observations agree with prior reports showing that MitoQ lowers mitochondrial ROS burden and lipid peroxidation in models of metabolic liver injury, obesity, and insulin resistance, while SS-31 has been shown in other organ systems and metabolic injury models to preserve cristae architecture, cardiolipin integrity, and respiratory efficiency [33,34]. In this context, our side-by-side comparison is particularly informative because relatively few studies have directly compared a redox-targeted mitochondrial antioxidant with a membrane-stabilizing mitochondrial peptide in the same MASLD model.

As hepatocyte apoptosis and loss are critical steps in the development from simple steatosis to steatohepatitis and fibrosis, protection against these processes is important [35]. Both MitoQ and SS-31 effectively decreased Bax expression, cytochrome c release, and the levels of cleaved caspase-3, while increasing that of Bcl-2, indicating strong anti-apoptotic effects. Of particular interest was that SS-31 appeared to pull ahead significantly in inhibiting the apoptotic signaling driven by hepatocytes, indicating the significance of maintaining mitochondrial membrane integrity as an effective gatekeeper for escaping into intrinsic apoptosis. This anti-apoptotic pattern is in line with previous work showing that mitochondrial membrane destabilization is a key upstream event in lipotoxic hepatocyte death. Studies investigating melatonin, resveratrol, N-acetylcysteine, and mito-targeted antioxidants have similarly reported reductions in Bax/Bcl-2 imbalance and caspase activation, suggesting that attenuation of mitochondria-mediated apoptosis is a common mechanism underlying hepatoprotection in steatotic liver disease [36] [37]. The somewhat stronger anti-apoptotic profile of SS-31 in the present study may reflect its direct action on mitochondrial membrane integrity, which could confer a more proximal blockade of intrinsic apoptotic signaling.

The strong relationship between mitochondrial injury, free radicals and inflammation are the major driving force for hepatic fibrosis in MASLD [20]. Mice consuming a HFHF diet activated inflammatory and fibrotic mechanisms, including NF-κB, NLRP3 inflammasome, α-SMA and TGF-β1—suggesting evidence of inflammation/scarring at the tissue level. MitoQ and SS-31 treatment markedly attenuated these inflammatory and fibrogenic signals, resulting in less collagen accumulation and better organized liver architecture. The suppressive effect of SS-31 was slightly superior to that of CoQ10 against the reduction of α-SMA, indicating a modest enhancement of anti-fibrotic action. These findings identify a key target for normalization that, when achieved, is sufficient to silence hepatocyte-driven inflammation and fibrosis and break the self-reinforcing circuit by which MASLD progresses. These results are supported by previous studies showing that oxidative stress-sensitive pathways such as NF-κB and the NLRP3 inflammasome are tightly linked to stellate cell activation and fibrogenesis in MASLD [38]. Similar anti-inflammatory and anti-fibrotic effects have been observed with mitochondria-protective interventions and antioxidant compounds in diet-induced steatohepatitis models, including reductions in TGF-β1 signaling, α-SMA expression, and collagen deposition [39]. Our findings therefore strengthen the concept that mitochondrial protection can influence not only steatosis, but also the inflammatory-fibrotic transition that underlies disease progression.

Insulin resistance, an additional MASLD hallmark, is closely associated with mitochondrial ROS production excess together with lipid accumulation and chronic inflammation. As found in our research, decreased levels of p-IRS1, p-Akt and GLUT2 in HFHF-fed mice revealed impaired glucose metabolism. These pathways were ameliorated by co-administration of MitoQ and SS-31, which led to amelioration in fasted glucose and reduction in HOMA-IR scores, indicating reduced insulin resistance. A concomitant restoration of mitochondrial structure and redox state was enough to reverse hepatic insulin resistance, further supporting the notion that a healthy pool of mitochondria is vital in the preservation of whole-body metabolic homeostasis. This observation is also in agreement with studies showing that improved mitochondrial efficiency and reduced oxidative stress are associated with restoration of insulin receptor signaling in liver and peripheral tissues. Similar improvements in IRS1/Akt signaling and glucose handling have been described following treatment with antioxidant or mitochondrial-supportive interventions in experimental obesity and fatty liver disease, indicating that the metabolic benefits observed here are biologically plausible and therapeutically relevant [40].

The separable but coordinated effects of MitoQ and SS-31 demonstrated here provide important insight into the mechanisms by which mitochondria-targeted therapies can be used to prevent MASLD. MitoQ acts in essence as a redox-regulating agent, stimulating the liver antioxidant defensing system and decreasing oxidative damage, whereas SS-31 seems to limit mitochondrial structural destabilization and apoptosis. These two aspects of the mitochondrial condition are targeted overall. Thus, both targeted therapies may have therapeutic benefits together greater than the sun of each alone. This mechanistic distinction is important when interpreted alongside the broader antioxidant literature. Conventional antioxidants such as vitamin E, polyphenols, and general ROS scavengers may improve some biochemical or histological endpoints, but their effects are often limited by poor mitochondrial specificity. In contrast, MitoQ and SS-31 act within the organelle most critically involved in MASLD pathogenesis, which may explain the broader spectrum of benefit observed in the present study. These data also raise the possibility that redox-targeted and membrane-stabilizing mitochondrial therapies may be complementary rather than redundant.

The application of aged female mice in this study provides significant translational relevance. Post-menopausal women are especially at risk for metabolic liver diseases, in part because of aging-related mitochondrial dysfunction, impaired antioxidant potential and enhanced insulin resistance. The capacity of MitoQ and SS-31 to alleviate mitochondrial dysfunction and enhance liver health in this model highlights their possible use in comparable at-risk human cohorts. This aspect of the study adds value because many preclinical MASLD investigations are performed predominantly in young male rodents, which may not fully capture the metabolic and hormonal context relevant to older women [41]. By using aged female mice, the present model more closely reflects a clinically vulnerable population in whom mitochondrial decline, estrogen deficiency, and metabolic stress may interact to accelerate liver injury. Accordingly, the translational relevance of mitochondria-targeted therapies may be particularly strong in this subgroup.

In summary, our results support the concept that MASLD is a mitochondria-centered metabolic disease. Once the anatomic and functional integrity of mitochondria is lost, then a sequence of events that includes oxidative stress, apoptosis occurs followed by inflammation, fibrosis and metabolic imbalance. MitoQ and SS-31 act directly at these origins of dysfunction to counter them, thereby simultaneously antagonizing multiple critical instigators of disease progression, providing a rational mechanism-based therapeutic approach. However, the present study has some limitations. Although we evaluated a broad panel of biochemical, histopathological, and molecular markers, the mechanistic assessment primarily relied on serum biochemistry, histological analysis, and Western blot–based profiling. Future studies incorporating additional functional approaches, such as mitochondrial respiration assays, more detailed ultrastructural analyses, and metabolic flux assessments, would further strengthen mechanistic interpretation of the protective effects observed here. In addition, although the experimental design included untreated control, HFHF-fed MASLD, and treatment groups, it did not include control animals treated with MitoQ or SS-31 alone. Inclusion of such groups would have provided additional insight into the baseline physiological effects of these mitochondria-targeted interventions under non-MASLD conditions. While this was beyond the scope of the present study, it remains an important consideration for future investigations and would further strengthen interpretation of treatment specificity. Subsequent work should strive to optimize dosing strategies, based on age and sex specific factors contributing to disease risk and treatment response as well as evaluate long-term outcomes and potential synergistic effects of combination therapy. From a clinical perspective, although these findings remain preclinical, they support further investigation of mitochondrial therapeutics as adjunctive or disease-modifying strategies in MASLD. Future studies should compare these agents with currently investigated metabolic and anti-inflammatory therapies, evaluate combination regimens, and determine whether benefits on mitochondrial quality translate into durable histological and metabolic improvement in human disease.

## Conclusion

Mitochondrial-targeted interventions reverse diet-induced hepatic steatosis in old female mice. MitoQ mainly boosts mitochondrial antioxidant system, and SS-31 maintains the structure of the mitochondria and inhibits apoptosis. Both agents improve hepatic inflammation, fibrosis and insulin resistance highlighting mitochondria as an important therapeutic target. These results offer a strong case for considering combination mitochondrial therapeutics to combat the multifaceted pathophysiology of MASLD and may have therapeutic relevance for aging and metabolically susceptible populations.
